# Longitudinal follow up of serological response in children treated for Chagas disease

**DOI:** 10.1371/journal.pntd.0007668

**Published:** 2019-08-29

**Authors:** Guillermo Moscatelli, Samanta Moroni, Facundo García Bournissen, Nicolás González, Griselda Ballering, Alejandro Schijman, Ricardo Corral, Margarita Bisio, Héctor Freilij, Jaime Altcheh

**Affiliations:** 1 Parasitology service Ricardo Gutiérrez Children´s Hospital, Buenos Aires, Argentina; 2 Multidisciplinary Institute for Research of Pediatric Diseases- CONICET-GCBA, Buenos Aires, Argentina; 3 Molecular Biology Laboratory of Chagas disease, INGEBI-CONICET, Buenos Aires, Argentina; Sacro Cuore Hospital, ITALY

## Abstract

**Background:**

Evaluation of therapeutic response in chronic Chagas disease is a major challenge, due to prolonged persistence of *Trypanosoma cruzi*-specific antibodies, lack of sensitivity of parasitological tests, and need for long-term follow-up to observe negative seroconversion of conventional serological tests (CS). The objective of this study was to evaluate F2/3-ELISA serology, a promising early biomarker of therapeutic response, and *T*.*cruzi* Polymerase chain reaction (PCR) for *T*. *cruzi* Deoxyribonucleic acid (DNA), for neonatal diagnosis and evaluation of parasitemia after treatment.

**Methods:**

Prospective cohort study, with three-year clinical, serological and parasitological follow-up of pediatric Chagas disease patients treated with benznidazole. Serology was evaluated by Enzyme-Linked ImmunoSorbent Assay (ELISA), Indirect hemagglutination (IHA) and F2/3-ELISA; Parasitemia by microhematocrit (MH) and PCR.

**Results:**

A cohort of 107 pediatric patients treated with benznidazole was enrolled in the study. ELISA and IHA were initially reactive in 100% of patients, F2/3-ELISA serology was reactive in 80% (86/107) and 91% (97/107) had detectable parasitemia. Seventy-six (71%) patients completed at least 36 months of serological follow up after treatment. Although a similar decreasing linear trend was observed for all serological tests, F2/3-ELISA presented earlier, age dependent, negative seroconversion compared to CS. All patients reaching undetectable CS titers had previously seroreverted by F2/3-ELISA. All patients with persistently decreasing antibody titers had negative PCRs throughout the follow up period. No new cardiological lesions were observed during the 3 years follow-up period.

**Conclusions:**

The data reported here, using CS, F2/3 ELISA and PCR provide support for the efficacy of benznidazole in congenital Chagas diseases. These results provide support for scaling up of screening, diagnosis and access to benznidazole treatment.

**Trial registration:**

ClinicalTrials.gov 0028/04 in the Research Council, Secretary of Health Buenos Aires city Goberment.

## Author summary

Evaluation of therapeutic response in chronic Chagas disease is a major challenge, particularly in the early post-treatment phase, due to prolonged persistence of *Trypanosoma cruzi*-specific antibodies and lack of sensitivity of available parasitological tests. The main limitation in evaluating Chagas disease treatment response stems from the need for long-term follow-up to observe negative seroconversion of conventional serological tests. New biomarkers of cure are needed. We evaluated F2/3-ELISA, a promising early serological marker of therapeutic response, and *T*.*cruzi* PCR for *T*.*cruzi* DNA, for neonatal diagnosis and evaluation of parasitemia after treatment. F2/3-ELISA and PCR proved to be excellent early markers of treatment response that correlate with ELISA and IHA but can identify treatment response or failure at much earlier timepoints. This information can help design future paediatric clinical trials in Chagas disease.

## Introduction

Chagas disease (CD), or American trypanosomiasis, caused by *Trypanosoma cruzi* affects an estimated 6–7 million people in Latin America [1], and has recently evolved into a global health concern due to migration [2]. *T*.*cruzi* has a broad range of hosts, including wild and domestic animals, and is primarily transmitted by infected haematophagous Triatominae bugs. However, due to improved vector control and migration of infected people to urban areas without the vector, presently the most common infection route is congenital [3].

CD features an initial acute phase with high parasitaemia. Clinical symptoms are variable and decline spontaneously after some weeks but the majority of subjects are asymptomatic. During this phase of the infection appropriate treatment can eliminate the parasite, leading to rapid negative seroconversion. Patients who remain untreated will progress into an “indeterminate stage” with intermittent parasitemia but no overt clinical manifestations and, eventually, into a chronic phase characterized by low level parasitaemia and presence of anti-*T*.*cruzi* antibodies.

The majority of chronic patients remain in the indeterminate stage, but approximately 30–40% eventually develop cardiac or gastrointestinal complications in the long term [4]. Evaluation of therapeutic response in chronic CD is a major challenge due to prolonged persistence of *T*.*cruzi*-specific antibodies and low sensitivity of available parasitological tests [5,6]. Furthermore, no test currently in use (e.g. ELISA, IHA, PCR, etc) has been validated for long term follow up of patients, as they were initially developed for diagnostic purposes.

Use of conventional serological methods (CS) such as ELISA and IHA for follow-up after treatment is widespread but has never been fully validated, and has proven unsuccessful in demonstrating treatment response in most patients as total anti-*T*.*cruzi* antibodies may take years to become negative [7]. However, pediatric trials for therapeutic markers are largely lacking.

The main limitations in evaluating treatment response for CD stems from the need for long-term follow-up (years to decades) to observe negative seroconversion of CS tests. In this context, new markers of cure are needed. Alternative early markers of cure have been suggested, such as decrease of total anti-*T*.*cruzi* antibody titers (i.e. instead of negative seroconversion) or use of non-conventional serological techniques [8,9] such as specific lytic anti-α-Gal antibodies known as anti-F2/3 antibodies [5,10]. Similarly, polymerase chain reaction against *T*.*cruzi*-DNA (PCR), has been proposed as a sensitive and specific method to detect parasitemia in newborns [11,12] and during follow-up after treatment [12,13].

The aim of this study was to describe, in a cohort of infants and children, serological and parasitological response during a three-year follow-up after treatment with benznidazole for CD.

## Materials and methods

### Ethics statement

Study protocol was reviewed by Research & Teaching Committee and the Bioethics Committee of the “Ricardo Gutiérrez” Children´s Hospital, and the Secretariat Committee for Research Involving Human Subjects, World Health Organization (Geneva, Switzerland). Written informed consent was obtained from legal guardians of the minors, as well as patient consent or assent, as appropriate.

### Study design

Prospective, cohort study of pediatric CD patients treated with benznidazole (S2).

### Patients

Infants and children living in the City of Buenos Aires, Argentina, a non-endemic area for CD, diagnosed at the Parasitology and Chagas Service, Children’s Hospital “Ricardo Gutierrez” between 09/2003 and 10/2007. Patients with *T*.*cruzi* infection, less than 20 years old and previously untreated for CD were enrolled in the study if they had no cardiovascular, hepatic, neurologic, endocrine, or other major systemic diseases and were not immunocompromised.

CD was diagnosed as positive parasitemia by microhematocrit (MH) in patients under 8 months [14] or, in older patients, if they had at least two reactive CS, ELISA (CS-ELISA), indirect hemagglutination (IHA), or particle agglutination. Sera obtained by centrifugation for serological assays were kept at -20°C until analyzed.

F2/3-ELISA Chemiluminescent ELISA for detection of anti-F2/3 antibodies was performed purified using F2/3 glycoconjugates [15] according to Almeida et al.

Serological results were expressed as the difference between the optical density value recorded for each serum sample and the cut-off value of the assay.

Detection of *T*.*cruzi*-DNA by PCR was done in blood (2 mL) mixed with EDTA-guanidine buffer [12,16].

### Clinical and biochemical evaluation

Serology by CS-Elisa, IHA and F2/3-ELISA, were done at the initial visit and repeated at 7, 30 and 60 days of drug therapy, every 3 months during the first-year post-treatment and every 6 months thereafter for the 36 months post treatment. MH for parasitemia was performed weekly until negative in subjects with positive MH at diagnosis; PCR was carried out at diagnosis and at 7, 30 and 60 days of drug therapy, and 3, 6, 12, 24 and 36 months post treatment.

Although it was planned as an observational study with a 36 months follow-up, in a cohort of patients we use available data along a longer follow-up, completing 96 months.

Laboratory evaluation (haematology, hepatology and renal function biochemical tests, and Pregnancy test for females of childbearing potential.), electrocardiogram, and echocardiogram were performed before and, at the end of treatment; Clinical evaluation was done at every visit, and electrocardiogram and echocardiogram were repeated yearly during follow-up.

Treatment: benznidazole (100-mg tablets) was prescribed at 5–8 mg per kg, in two or three daily doses for 60 days. Infants’ doses were provided as fractionated tablets prepared by a research pharmacist and administered with milk. Medication was provided in monthly batches and compliance was assessed by counting the remaining tablets at each visit. Adverse drug reactions (ADRs) were evaluated through laboratory tests, clinical interrogation and physical examination.

### Statistical analysis

Continuous variables are presented as means with CI95% or medians and interquartile range, and categorical variables as percentages.

The disappearance kinetics of serum antibodies were analyzed using survival analysis. Significance levels for analysis were 0.05. Analyses were performed with R software v3.0 (R Core Team 2018. R Foundation for Statistical Computing, Vienna, Austria. https://www.R-project.org/).

## Results

**Fig 1:**
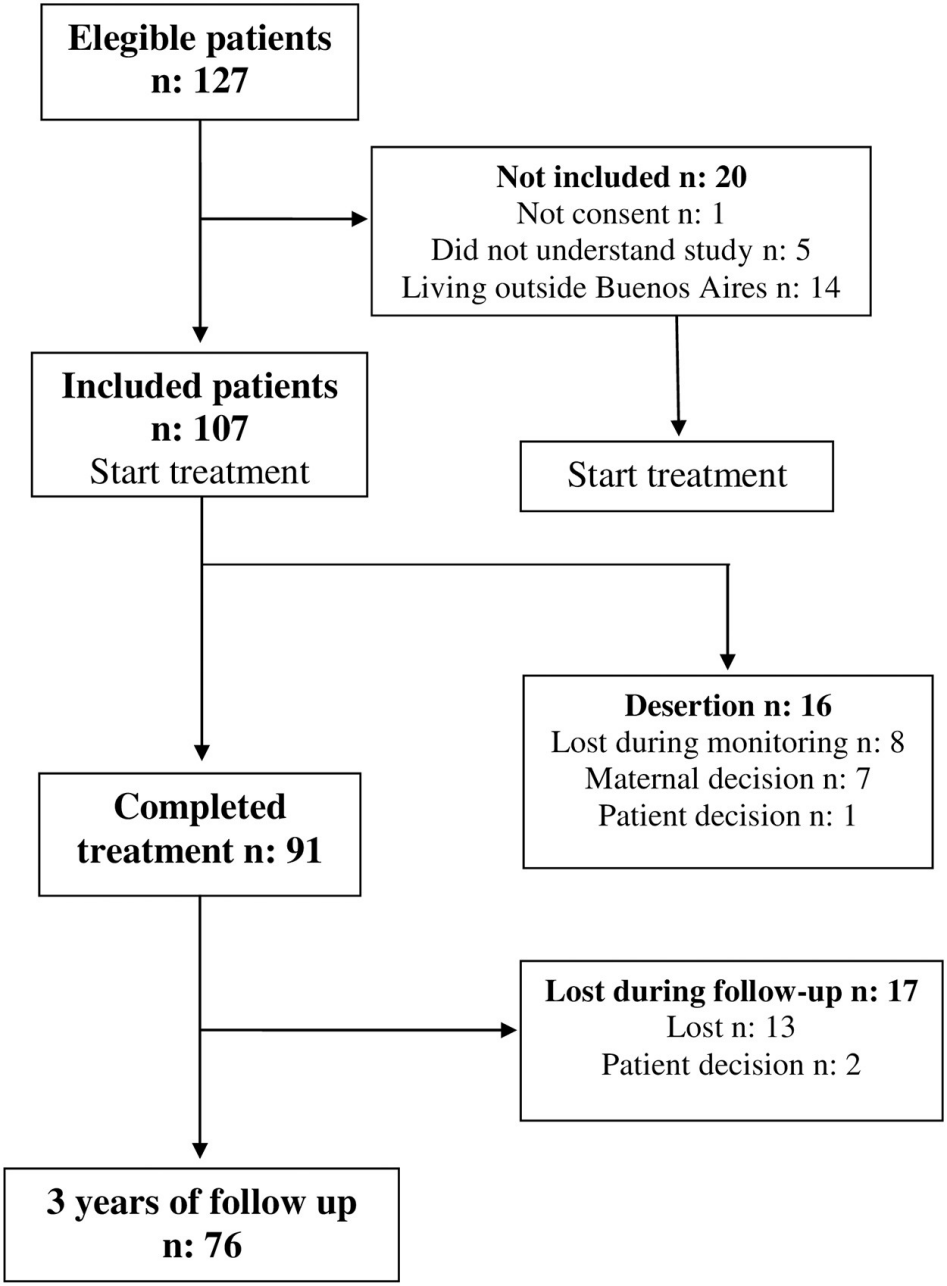
Flow diagram of children enrolled in the study and information about sides effects.

Out of 127 pediatric Chagas disease patients screened, 107 patients were enrolled in the study (Fig 1).

Demographic data are shown in Table 1.

**Table 1:** Demographic data.

Residence in Buenos Aires	n = 107 (100%)
Median patient age	6.9 years (range: 10 days-19 years)
Route of infection:	
Transplacental	67.3%
Vectorial	4.7%
Undefined	28%
Maternal origin of congenitally infected infants:	
Argentina	59.8%
Bolivia	29%
Paraguay	10.3%
Brazil	0.9%

All patients were asymptomatic at diagnosis, with normal electrocardiogram and echocardiogram, and no Chagas disease-associated pathology.

Treatment: Mean benznidazole dose was 6.4 mg/kg per day (range 5–8 mg/kg) in 2 divided doses (n = 76) or 3 divided doses (n = 31). Mean treatment length was 60 days (CI_95_ 59–61). We observed good compliance on the basis of tablet counts and medication log review. A total of 91/107 (85.0%) enrolled patients completed drug treatment. Benznidazole was well tolerated and ADRs were mild, not requiring treatment suspension [17].

Patient retention during long term follow up was as follows: 92 (86%) at 3 months after treatment, 86 (80.3%) at 12 months, 82 (76.6%) at 18 months, 80 (74.7%) at 24 months, 76 (71%) at 30 months and 76 (71%) at 36 months (Fig 1). In addition, we have completed follow-up beyong the limit of the protocol for clinical reasons for 41 (38%) subjects at 72 months after treatment.

### Parasitological studies

Parasitemia was positive in 97/107 (90.6%) patients before treatment (84 by PCR, 8 by both PCR and MH, and 5 by MH test). All 13 patients younger than 8 months of age had positive MH, and 8/8 (100%) for whom PCR samples were available also had positive PCR (PCR samples were not available for 5 patients with positive MH). (Table 2).

**Table 2:** PCR results during treatment follow up. Patients who became negative remained negative. Positive results are for patients who had not became negative before.

Time of follow up	n	Positive result	Percent (CI_95_)
0	102	92	90.2 (83.9–95.2)
7 days	94	38	40.4 (29.4–48.9)
30 days	91	4	4.4 (1.1–9.3)
60 days	88	1	1,1 (0.5–8.9)
5 months	81	2	2.5 (0–4.5)
8 months	78	2	2.6 (0.7–9.0)
12 months	77	2	2.6 (0.7–9.1)
24 months	74	1	1.3 (0.3–8.2)
36 months	74	1	1.3 (0.3–8.2)

In patients younger than 8 months MH test rapidly became negative after start of treatment; in 10/13 (76.9%) MH was negative at 7 days of treatment (p<0.001, Fisher’s exact test) and the remaining 3 positive patients became MH negative within 30 days of treatment. Parasitological response was evaluated by PCR in 102/107 patients (5 infants did not have enough sample for PCR); PCR remained positive in 38/94 (40.4%) patients at 7 days of treatment, 4/91 (4.4%) at 30 days and 1/88 (1.1%) at end of treatment (Fisher’s exact test, p<0.001 for all comparisons against baseline) (Table 2, PCR results during treatment follow up. Patients who became negative remained negative. Positive results are for patients who had not became negative before).

### Serological studies

CS-Elisa and IHA were initially reactive in 107/107 (100%) of patients at diagnosis; F2/3-ELISA was reactive in 105/106 (81.1%) (1 patient had no F2/3-ELISA samples at diagnosis).

**Fig 2:**
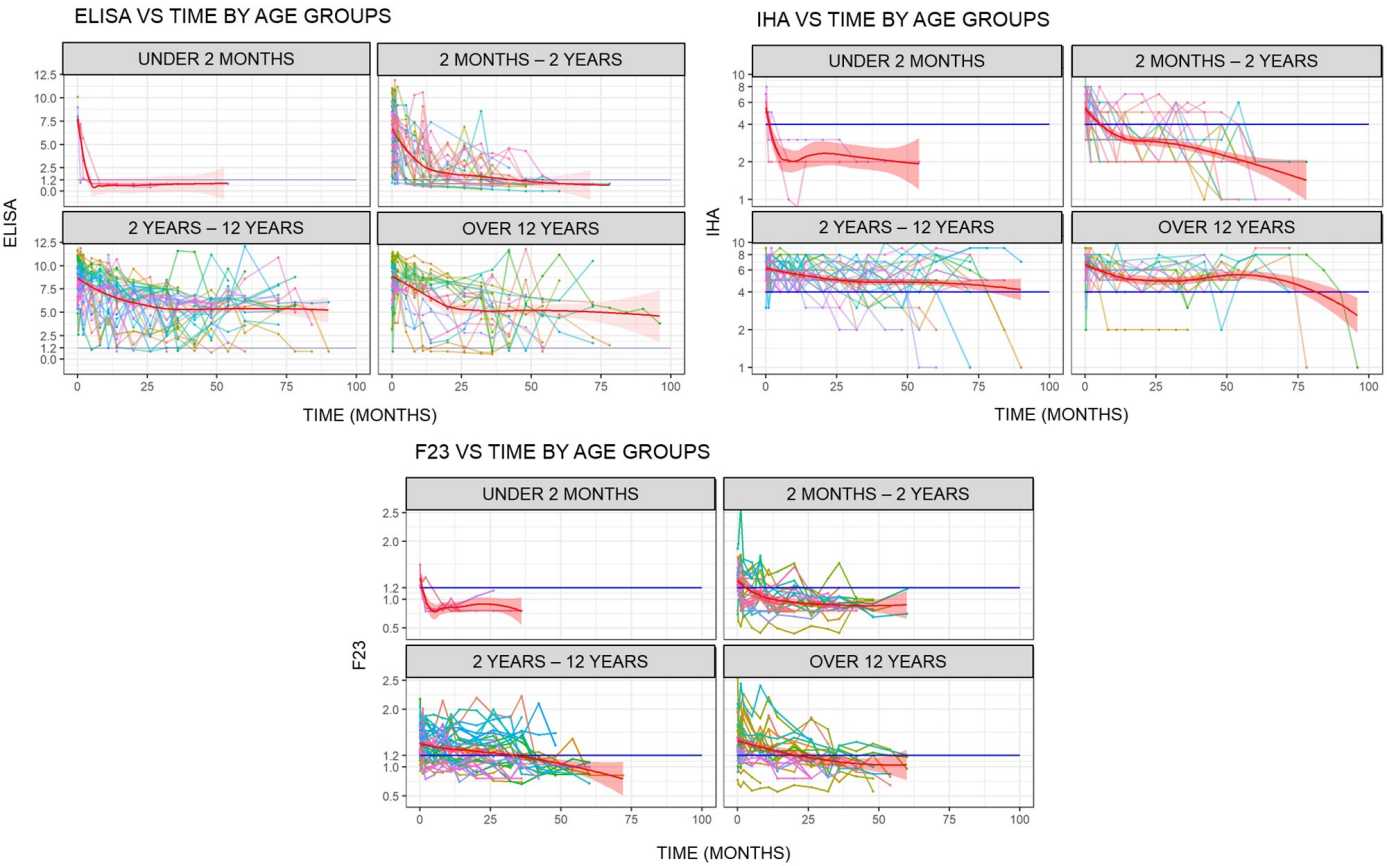
Individuals serological profiles versus time: For ELISA; for IHA, and for F2/3. Thick red line = smooth regression of the data. Horizontal blue line = cut value.

Seventy-six patients completed at least 36 months serological follow up by both CS and F2/3 ELISA. CS-ELISA, CS-IHA and F2/3-ELISA values were analyzed at each time point during follow-up, stratified by patient age (Fig 2).

**Fig 3:**
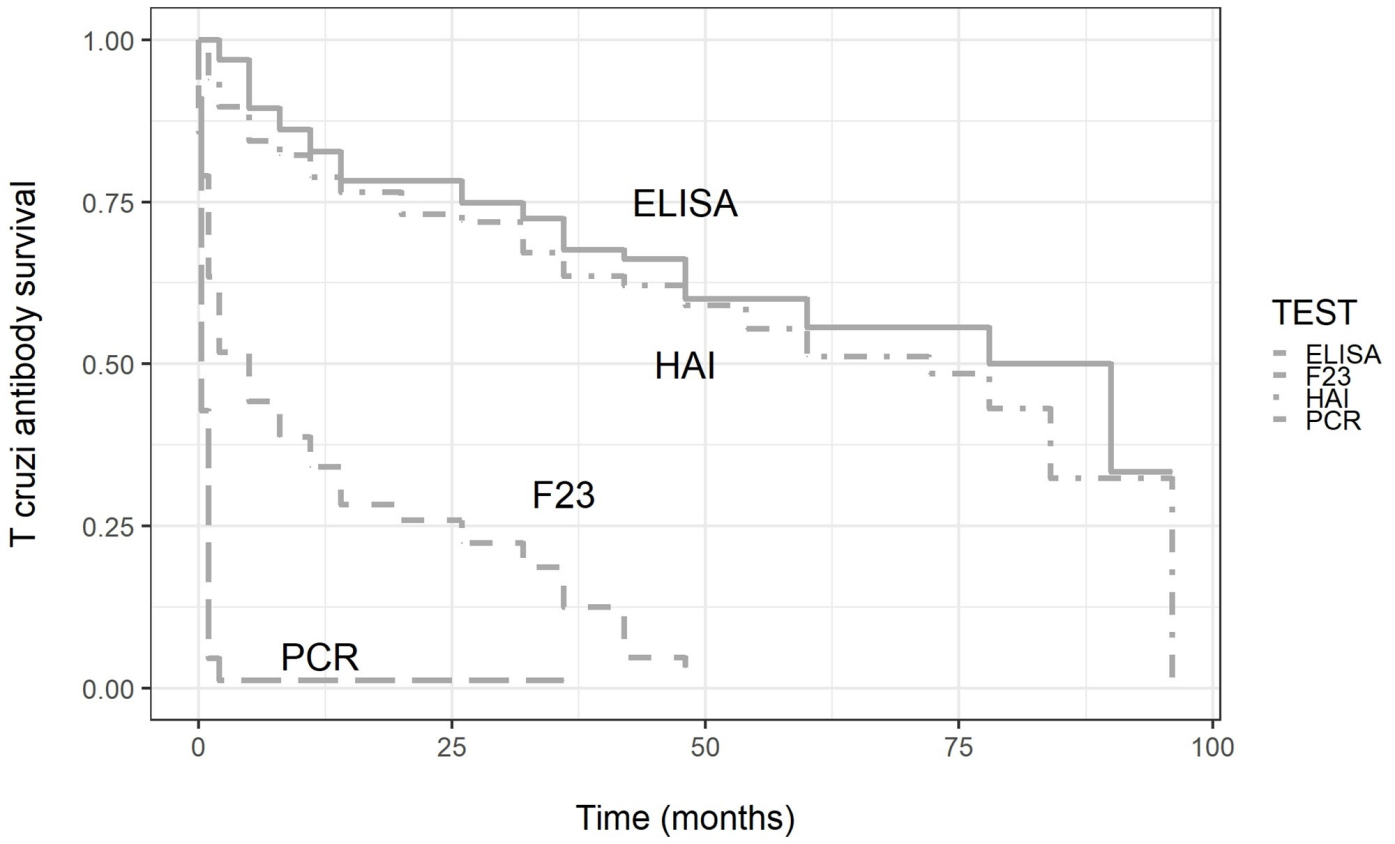
Survival curve time to negative serology or PCR.

A progressive reduction was observed for CS-ELISA, CS-IHA and F2/3-ELISA (Fig 3).

**Fig 4:**
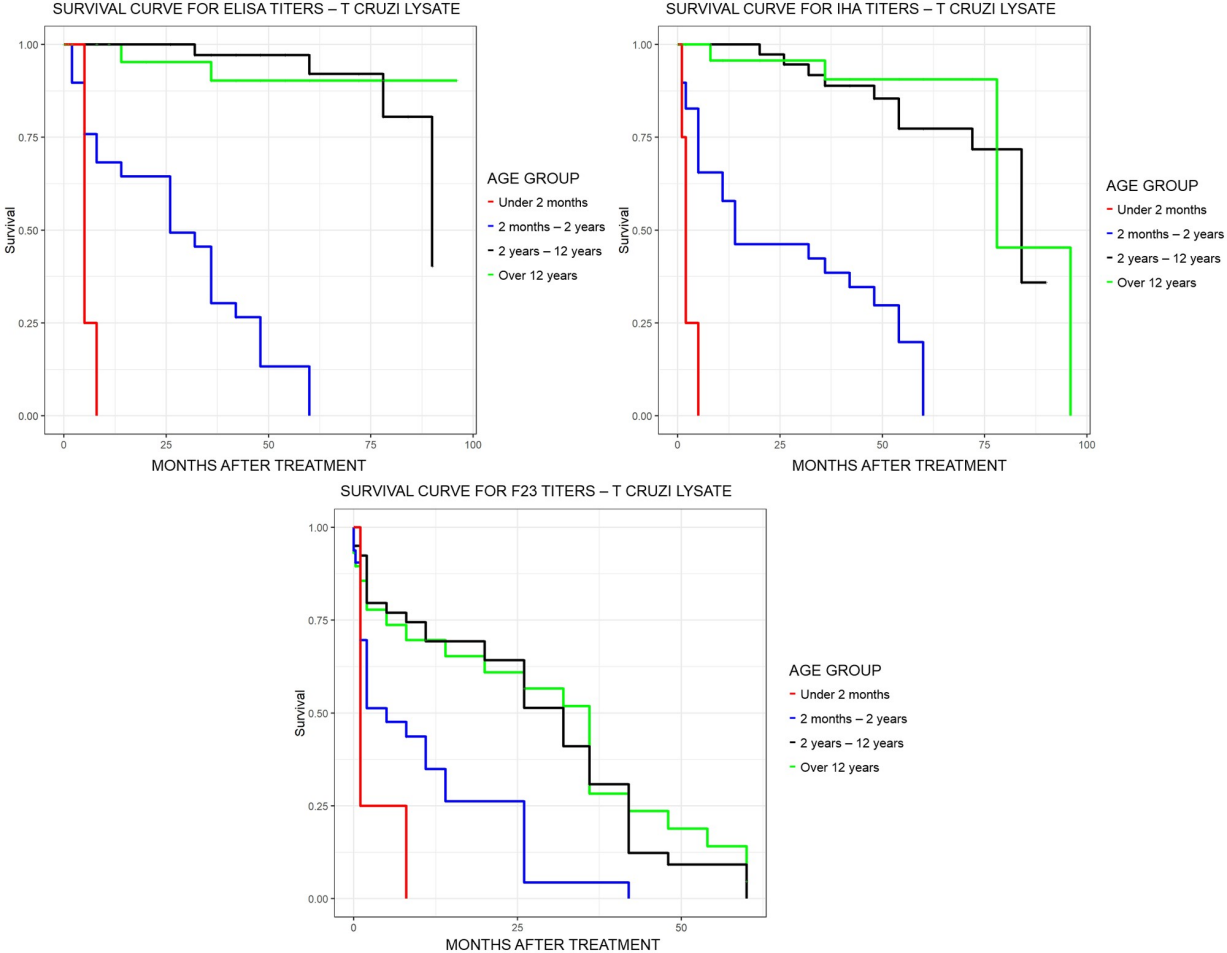
Survival curve time to negative serology by age group: For ELISA; for IHA; and for F2/3.

Although kinetics showed a similar decreasing linear trend in all tests, F2/3-ELISA presented earlier negative seroconversion than CS (Table 3, Fig 4).

**Table 3:** Median of survival time of antibody anti-T. cruzi.

Age group	N	IHA Median [CI_95_] (months)	N	Tc-ELISA Median [CI_95_] (months)	N	F2/3-ELISA Median [CI_95_] (months)
0 to 2 m	6	2 (1-NA)	6	5 (5-NA)	5	1 (1-NA)
3m to 2y	32	14 (11–54)	32	26 (14–42)	32	5 (2–26)
3y to 12y	40	84 (84-NA)	40	90 (90-NA)	40	32 (26–36)
13y to 19y	29	78 (78-NA)	29	NA	29	36 (14–48)

IHA: indirect hemagglutination comercial kit; Tc-ELISA: comercial ELISA kit using Trypanosoma cruzi lysate antigen-coated microplates; F2/3-ELISA: in-house chemiluminescent ELISA using F2/3 antigen-coated microplates; m: months; y: years; NA: not available.

A total of 84.4% (38/45) patients under 2 years that completed 36 months of follow up with both CS-ELISA and CS-IHA, 29 seroconverted (p<0.001 compared to baseline, Chi-squared test), and 9 had decreases in antibody titers but failed to reach negative values during this follow up period. Ten patients less than 2 years old were lost to follow up before negative seroconversion.

Children over 2 years of age (62/107) showed a persistent decrease of antibodies titers in CS; 4 patients became negative, all later than 32 months (Median time to conversion: 72 months, range 32–90). All patients with persistently decreasing antibody titers (even if still positive) also had negative PCR throughout the follow up.

Out of 105 patients with initial positive F2/3-ELISA antibodies testing, 66 completed follow up with this method. In this population 48/66 (72.7%) became negative during follow up (p<0.001, Chi-squared test): 15 (33.3%) became negative at end of treatment, 9 (13.3%) at 6 months post-treatment, 5 (11.1%) at 9 months, 4 (8.9%) at 26 months, 9 (20%) at 36 months and 6 (13.4%) at 42 months. The median age of patients who became negative on F2/3-ELISA was 6,7 years (range: 10 days-19 years). On the other hand, the median age of the patients who remained with the positive F2/3-ELISA throughout the follow-up was 11.3 years (range: 4.5 years-19 years).

All patients reaching undetectable CS titers had previously become negative by PCR and F2/3-ELISA.

No cardiac alterations by electrocardiogram or echocardiogram were observed during follow-up of any of the patients in the cohort.

A total of 74/76 (97.4%) patients had persistently negative PCR results at 3 years follow-up. Two patients (3 months and 7 years old) showed persistently positive PCR and serology in all samples during follow-up (Table 2). The mother of the 3 months old boy eventually acknowledged that she had not administered the treatment to her infant, and a new round of benznidazole treatment was attempted with good response. The other 7 years old child received benznidazole treatment appropriately and therefore was considered as treatment failure; a new treatment with nifurtimox 10 mg/k/day for 60 days was indicated, with good response. Both children showed negative PCR and a decrease in CS antibody titers in the 3 years of follow-up after the second treatment.

## Discussion

Two drugs are available for the treatment of CD: benznidazole and nifurtimox. The effectiveness of these drugs, especially in the chronic stage of infection, is still a topic of debate due to inconsistent results and a lack of early biomarkers of treatment response [18,19].

The treatment goals for *T*.*cruzi* infection are to eliminate the parasite and to decrease the probability of clinical progression of the disease.

Several studies have suggested that the etiological treatment of CD leads to negative results in non-conventional serological tests and/or the prevention of electrocardiographic and clinical changes related to disease progression [19,20,21,22].

However, other studies are contradictory and indicate that when treatment is administered during the chronic phase of the disease, the parasite is not completely eliminated, and the progress of the disease is not interrupted; therefore, the complications of the infection are not prevented [23,24,25,26,27]. Controversies still exist regarding the real effects of the drugs used in human treatment on the clinical progression of CD, particularly in adults during the chronic phase of the infection.

These contradictions are most likely caused by the use of different treatment regimens and/or post-treatment evaluation protocols [28].

Here we present the parasitological and serological follow up of a large cohort of infants and children, mainly infected by the transplacental route, treated with benznidazole in the acute and early chronic phase of the infection. These patients were asymptomatic and without cardiological involvement at the time of diagnosis and treatment and therefore treatment had no demonstrable immediate clinical benefit. During the 3 years follow-up period, no new cardiological lesions were observed. Further studies with longer follow-up are needed to confirm these results. In this context, identification and validation of biomarkers in our large cohort of congenital cases will enable the development of much needed new and improved treatments.

Our results also show a high rate of therapeutic response, as measured by different serological tests and by *T*.*cruzi*-PCR. Treatment was well tolerated with mild adverse events as was reported in a previous publication [17].

There are several choices to diagnose Chagas disease and monitor drug efficacy. MH is the direct parasitological method of choice to identify congenital infection in newborns and infants because of its high sensitivity and the small amount of blood needed [6]. Evaluation of drug efficacy, on the other hand, commonly relies on negative conversion of parasitological tests that detect anti-*T*.*cruzi* antibodies [29]. However negative seroconversion by conventional serology can take years, or even decades, after treatment in older children and adults and is therefore not an adequate treatment response endpoint [10,30].

While PCR may be more sensitive than MH in some centers, the current lack of standardization of the method across centers is a still unresolved issue. Furthermore, actual rate of false positives is still under debate and may vary among testing laboratories (and PCR methods used), and other issues such as cost and instrument availability, and technical skills conspire to limit the use of this technique at the moment.

In our cohort, most of the treated children younger than 2 years (74.3%) rapidly became negative by MH and/or PCR, CS and F2/3-ELISA with an excellent correlation among all of them, supporting their usefulness as markers of parasiticidal effect in this age group. Children older than 2 years of age showed lower incidence of seroconversion but widespread CS and F2/3-ELISA seroreduction coupled to early negative PCR results after treatment. Significant reductions of *T*.*cruzi* antibody levels have been hypothesized to be a predictor of future negative seroconversion [22,30].

Previous published placebo-controlled trials in children [31,32] showed reductions of approximately 20% in *T*.*cruzi* antibody levels by CS-ELISA at 12 months follow up in treated pediatric patients. In contrast, placebo-treated patients showed persistent reactive serology without modifications of antibody titers. A significant difference between the treatment and placebo groups was apparent as early as 6 months after treatment. This was also observed in a historical series of treated patients with acute vectorial infection where the treated group showed a persistent decay in antibodies, while the untreated group showed no significant changes in antibody titers and persistence of positive parasitological tests during follow-up [33], suggesting that a decrease in antibody concentration is a marker of adequate treatment response.

Persistent positive serology after treatment, in subjects with conversion to non-reactive *T*.*cruzi*-PCR, could be justified by the permanence of an immune response generated by self-antigens. Heart proteins may exhibit cross-reactivity with parasite surface antigens, a phenomenon known as molecular mimicry [34]. In other infections, after treatment, antigens remain in phagocytic cells, dendritic cells and macrophages long after treatment, and immune memory cells can continue to produce antibodies and mount specific immune responses decades after the infection or vaccination. In addition, carbohydrate determinants present in gastrointestinal and pulmonary microflora could stimulate lymphocytes previously primed by *T*.*cruzi* epitopes, sustaining reactive serology in otherwise cured subjects [35].

Identification of novel and reliable post therapeutic markers is needed, such as surrogate markers to identify absence or reduction of parasite load, which should be quicker and more sensitive than seroconversion by conventional serology.

Using non-conventional serological techniques, i.e. highly sensitive and specific chemiluminescent ELISA using a purified trypomastigote glycoconjugate antigen (AT or F2/3 ELISA) [32], the time to negative seroconversion was significantly faster for AT/F2-3 ELISA than for the CS (ELISA, IHA).

In a previous study by our group [5], the kinetics of disappearance of conventional serology and anti-F2/3 antibodies were compared in 21 patients with congenital CD after receiving benznidazole treatment. In patients younger than 8 months, antibodies were undetectable by both conventional serology and F2/3-ELISA soon after treatment. In older infants a negative F2/3-ELISA result occurred earlier than for CS.

In our study these results were reinforced with a large cohort where F2/3-ELISA became negative earlier than CS-ELISA. Consequently, a negative F2/3-ELISA should be considered as a surrogate endpoint for assessment of positive response to treatment, particularly in those patients with prolonged time of infection. However, usefulness of F2/3 ELISA is limited due to the lack of standardized commercial kits which makes reproducibility of results difficult. Furthermore, the fact that a sizable proportion of patients are already negative at the start of the treatment decreases the population that may benefit from follow up with this method.

In an effort to obtain more sensitive parasitological assays, a *T*.*cruzi*-PCR strategy has been developed to detect *T*. *cruzi* DNA in blood samples. Thus, primers have been designed for the amplification of nuclear and kinetoplast *T*.*cruzi*-DNAs, both of which contain many repetitive sequences that are highly suitable for *T*.*cruzi*-PCR detection [36].

Several studies have shown that *T*,*cruzi*-PCR is a more sensitive parasitological marker for treatment failure than classical parasitological procedures [12,13,36,37,38,39].

Our study shows that PCR is an early marker of treatment response since PCR was negative in 98.5% of patients at the end of treatment, remaining negative during long term follow-up. These results are in line with previous reports from our group [12] and from other authors [11,13,36,40].

In our cohort of congenital cases, negative PCR strongly correlated with seroreduction or negative seroconversion by CS and F2/3-ELISA. This occurred in all patients in both the acute and early chronic indeterminate phase of infection.

PCR provides a helpful clinical tool for early detection of treatment failure. As an example, in 2 cases in our series, PCR remained positive after treatment and levels of T.*cruzi* antibodies remained unchanged by CS and F2/3 ELISA, suggesting treatment failure given that these patients lived in a non-endemic area (i.e. could not have suffered re-infection). The fact that the mother of one of these patients later acknowledged that she had not administered the medication further strengthens this point. The patients were later treated, one with nifurtimox and the other one with benznidazol at standard doses for 60 days, with good therapeutic response, and showed negative conversion of PCR and decrease in CS and F2/3 ELISA confirming that these tests are sensitive both to treatment response and treatment failure.

Our study has some limitations, including lack of very long follow ups (eg. decades) to confirm that treatment response, as measured by serology and PCR, correlates with decreased risks of cardiac involvement decades later. However, such prolonged follow up periods are technically impossible at this stage. The absence of an untreated control group in our cohort could be considered a limitation too, but, as mentioned before, there is extensive evidence showing that patients that are not treated continue to have positive PCR and CS-ELISA titers do not fall in time. This evidence has prompted laws and guidelines mandating treatment of pediatric Chagas disease, precluding the inclusion of an untreated cohort in pediatric studies (which would be considered unethical).

We concluded that the data reported here, used CS, F2/3 ELISA and molecular biology-based laboratory tools (i.e. PCR) to demonstrate efficacy of chemotherapy in pediatric CD. We believe it is vital to reinforce the need to screen all pregnant women living in or emigrating from endemic areas, in order to provide their newborns with an early accurate diagnosis for more successful treatment outcome [41].

Finally, these results provide support for the scaling up of diagnosis and access to standard regimens of benznidazole.

## Supporting information

S1 File This is the trial registry information. (PDF)

S2 File This is the final protocol of the study. (PDF)

S3 File Flow chart. (DOC)

S1 Checklist STROBE checklist. (DOC)
