# Supplementary material for: Longitudinal follow up of serological response in children treated for Chagas disease
Source: PLoS Negl Trop Dis. 2019 Aug 29;13(8):e0007668. doi: 10.1371/journal.pntd.0007668 (PMC6715178; doi:10.1371/journal.pntd.0007668)
Supplement: S1 File — (PDF) [file pntd.0007668.s001.pdf]

GOBIERNO DE LA CIUDAD DE BUENOS AIRES
SECRETARIA DE SALUD
CONSEJO DE INVESTIGACION EN SALUD

INFORME N° 82 -SS-CIS-2004

Buenos Aires, 7 de septiembre de 2004

MOTIVO: S/ Protocolo de Investigación "Marcadores de respuesta terapéutica en pacientes con infección por Trypanosoma cruzi".

Dr. Hector Freilij
Laboratorio de Parasitologia
Hospital de Niños Dr. R. Gutierrez

1.- Por la presente informamos a Ud. que el protocolo "Marcadores de respuesta terapéutica en pacientes con infección por Trypanosoma cruzi" ha sido aprobado en la fecha por el Consejo de Investigación en Salud y ha quedado registrado con el N° 0028/04 de acuerdo con lo establecido por la Resolución N° 1914-SS-04.

Sin otro particular, aprovechamos la oportunidad para saludarlo muy atentamente.

Dra. Margarita Vitacco
Presidente
Consejo de Investigación en Salud
