# Supplementary material for: Longitudinal follow up of serological response in children treated for Chagas disease
Source: PLoS Negl Trop Dis. 2019 Aug 29;13(8):e0007668. doi: 10.1371/journal.pntd.0007668 (PMC6715178; doi:10.1371/journal.pntd.0007668)
Supplement: S2 File — (PDF) [file pntd.0007668.s002.pdf]

Markers of Therapeutic response in congenital, paediatric and adult patients with trypanosoma cruzi infecion

Study Protocol Titel:

"Validation of F2/3 antibodies as Markers of Therapeutic response in paediatric and adult patients with trypanosoma cruzi infecion treated with benznidazole"

FINAL PROTOCOL DATE: 5 May 2003

TABLE OF CONTENTS **Page No**

1 GENERAL INFORMATION 4

1.1 PROTOCOL TITLE: 4
1.2 SPONSOR: 4
1.3 PRINCIPAL INVESTIGATOR: 4
1.4 CO-INVESTIGATORS: 4
1.5 TRIAL SITE: 5
1.6 NAME AND ADDRESS OF LABORATORY/INSTITUTION INVOLVED IN THE TRIAL 5

2 BACKGROUND: 6

3 TRIAL OBJECTIVES 7

3.1 PRIMARY OBJETIVE 7

4 TRIAL DESIGN 7

4.1 THE PRIMARY AND THE SECONDARY END-POINTS 7
4.2 DESCRIPTION OF THE TRIAL 7
4.3 CASE DEFINITION OF CHAGAS' DISEASE: 7
4.4 STUDIES DESCRIPTION 7
4.5 FOLLOW-UP 8
4.6 TIMETABLE FOR CLINICAL AND PARASITOLOGICAL ASSESSMENT 9
4.7 BLOOD COLLECTION AND SAMPLING SCHEDULE 10
4.8 CASE REPORT FORM (CRF) 10

5 SELECTION AND WITHDRAWAL OF SUBJECTS/PATIENTS 11

5.1 INCLUSION CRITERIA: 11
5.2 EXCLUSION CRITERIA 11
5.3 WITHDRAWAL CRITERIA 11

6 STUDY PROCEDURE 12

6.1 PATIENTS TREATMENT (WITHIN NORMAL PATIENT MANAGEMENT) 12
6.2 PRE TREATMENT 12
6.3 METHODS 12
6.4 STUDY FOLLOW-UP: 14
6.5 MEDICATION(S)/TREATMENT(S) PERMITTED 14
6.6 PROCEDURES FOR MONITORING PATIENT COMPLIANCE: 14

7 EFFICACY ASSESSMENT 15

7.1 SPECIFICATION OF EFFICACY VARIABLE AND PARAMETERS 15

8 STATISTICS 15

8.1 SAMPLE SIZE CALCULATION 15
8.2 DESCRIPTION OF STATISTICAL METHOD 15
8.3 SIGNIFICANCE LEVEL: 15

9 DATA HANDLING AND MANAGEMENT 16

10 ETHICAL ASPECTS 16

10.1 APPROVAL BY THE ETHICAL COMMITTEE 16
10.2 SUBJECT INFORMATION AND INFORMED CONSENT 16

10.3 CONFIDENTIALITY 16
11 REFERENCES 18
12 ABBREVIATIONS USED 19
13 TENTATIVE PLAN OF ACTION 19
13.1 TIME SCHEDULE IN MONTHS 19
14 CONSENT FORM (LOCAL LANGUAGE) 20
14.1 CONSENTIMIENTO DE PACIENTES ADULTOS PARA PARTICIPAR EN PROYECTO 20
Servicio Parasitología y Chagas. Hospital de Niños Ricardo Gutiérrez. *20*
14.2 CONSENTIMIENTO DE NIÑOS PARA PARTICIPAR EN PROYECTO 23
Servicio Parasitología y Chagas. Hospital de Niños Ricardo Gutiérrez. *23*

1 GENERAL INFORMATION

1.1 Protocol Title:

"Validation of F2/3 antibodies as Markers of Therapeutic response in paediatric and adult patients with trypanosoma cruzi infecion treated with benznidazole"

1.2 Sponsor:

World Health Organization,
Special Programme for Research and Training in Tropical Diseases (TDR), 20 Av. Appia, CH-1211, Geneva-27 Switzerland,
Hospital de Niños , Buenos Aires city Goverment

1.3 Principal Investigator:

Dr. ALTCHEH, JAIME MARCELO
Staff member of Servicio de Parasitología y Chagas
Hospital de Niños Ricardo Gutiérrez
Gallo 1330, C1425EFD, Ciudad de Buenos Aires, República Argentina
Tel +5411-4964-3093, email: jaltcheh@intramed.net.ar

Dr. FREILIJ, HECTOR LEON
Chief of Servicio de Parasitología y Chagas
Hospital de Niños Ricardo Gutiérrez
Gallo 1330, C1425EFD, Ciudad de Buenos Aires, República Argentina
Tel +5411-4964-3093, email: freilij@sinectis.com.ar
Fax: +5411-4962-3762

1.4 Co-investigators:

Dr. CORRAL, RICARDO SANTIAGO
Member of Research Career, National Research Council (CONICET), Argentina
Hospital de Niños Ricardo Gutiérrez
Gallo 1330, C1425EFD, Ciudad de Buenos Aires, República Argentina
Tel +5411-4964-3118, email: ricardocorral56@hotmail.com

BIANCARDI, MIGUEL ANGEL
Staff member of Servicio de Parasitología y Chagas
Hospital de Niños Ricardo Gutiérrez
Gallo 1330, C1425EFD, Ciudad de Buenos Aires, República Argentina
Tel +5411-4964-3093, email: biancardi@tutopia.com

Dr. LEVIN, MARIANO JORGE
Chief of Laboratorio de Chagas
Instituto de Ingeniería Genética y Biología Molecular (INGEBI)-CONICET
Vuelta de Obligado 2490. 2nd floor, Ciudad de Buenos Aires, República Argentina
Tel +5411-4783-2871, slafon@uba.ar

DR GUILLERMO MOSCATELLI
Hospital de Niños Ricardo Gutiérrez
Gallo 1330, C1425EFD, Ciudad de Buenos Aires, República Argentina

Dr Facundo Garcia Bournissen
Hospital de Niños Ricardo Gutiérrez

Gallo 1330, C1425EFD, Ciudad de Buenos Aires, República Argentina

1.5 Trial Site:

Ciudad de Buenos Aires, Argentina.

1.6 Name and address of Laboratory/Institution involved in the trial.

- Hospital de Niños Ricardo Gutiérrez (HNRG)
  Servicio de Parasitología y Chagas
  Gallo 1330, C1425EFD, Ciudad de Buenos Aires, República Argentina
  Tel +5411-4964-3093, email: freilij@sinectis.com.ar
  Fax: +5411-4962-3762

  Role of HNRG:
  - To provide patients health care
  - To provide working facilities for administration and logistic management.
  - Supply equipment (such as computers, microscopes).
  - To provide supporting staff (Laboratory technicians, Medical Doctors.).
  - To coordinate the work.

- Instituto de Ingeniería Genética y Biología Molecular (INGEBI) - CONICET
  Vuelta de Obligado 2490. 2nd floor, Ciudad de Buenos Aires, República Argentina
  Tel +5411-4783-2871, email: slafon@uba.ar

  Role of INGEBI:
  - To provide working facilities for biomolecular studies.

2 BACKGROUND:

According to a WHO scientific meeting held at Rio de Janeiro, Brazil, in 1998, parasiticidal drugs should be available for every *T. cruzi*-infected individual (1). Consequently, treatment now involves not only acute cases but also patients under the indeterminate and chronic stages of infection.

Presently, the accepted criterion for cure is the achievement of repeatedly negative outcome in conventional serology (CS), and eventually parasitological tests, after treatment. Traditional parasitological tests (xenodiagnosis, hemoculture) are of limited sensitivity and labor intensive. Therefore, of questionable value as a tool for evaluation of therapeutic response.

In those acutely (by vector-mediated transmission or by transplacental route) infected people who show good response to early treatment, CS becomes negative between 2 and 12 months post treatment (2). On the other hand, in pediatrics patients treated at the indeterminate phase of infection, CS becomes negative only after several years (3,4).

The reason for the persistence of reactive CS following treatment remains unclear. It has been postulated that this could be attributed to the occurrence of anti-idiotype serum antibodies raised by *T. cruzi* and/or by common antigenic epitopes shared by this parasite and saprophyte bacteria (5).

Consequently, there is a clear need for new serological markers that truly reflect the parasitological status of the patients.

In last years, it has been proven that: lytic antibodies, unlike antibodies detectable by CS, are associated with the presence of bloodstream trypomastigote forms in the host. Brazilian researchers have observed that patients treated during the indeterminate stage of infection presented the following pattern: - repeated negative hemoculture; - persistent reactive CS; and -progressive disappearance of lytic antibodies, (5,6). In view of the above, it has been postulated that the latter one constitutes another tool for monitoring viable parasites in indeterminate individuals presenting reactive CS (7).

Recently, several reports have identified that the F2/3 and some other *T. cruzi* antigens (ESAg, GPs 160, 75/90, T-DAF and 57/51, 24 kDa recombinant) are specific targets for lytic antibodies (8-10). These antibodies can also be analyzed through different immunoassays (ELISA, Western blot), which show a strong correlation with complement mediated lysis assay (CoML) (11).

In a preliminary study, the kinetics of disappearance of CS and anti-F2/3 antibodies was compared in 21 patients with congenital Chagas´ disease after receiving benznidazole treatment. A negative result for anti-F2/3 antibody detection significantly anticipated the disappearance of CS reactivity, particularly in those patients with prolonged time of infection.(12)

Amplification of specific sequences from T. cruzi DNA through polymerase chain reaction (PCR) allows detection of low concentrations of bloodstream parasites, showing higher sensitivity than traditional parasitologyc test. It has been reported as particularly helpful for patients at the indeterminate phase, which we were able to confirm in a small group of infants (13).

3 TRIAL OBJECTIVES

3.1 Primary Objetive

- Demonstration that reduction or disappearance of anti-F2/3 antibodies precedes CS test following treatment.

4 TRIAL DESIGN

4.1 The primary and the secondary end-points

Primary end-points:

Reduction of anti-F2/3 antibodies titers after treatment

Secondary end-points:

Negativization of anti-F2/3 antibodies after treatment.

4.2 Description of the trial

This is a multicenter prospective (longitudinal), open label, three year follow up of serological and parasitological evaluation of a cohort of Chagas´ disease patients treated with benznidazole.

Patients:

Children and adults attending the Servicio de Parasitología y Chagas (HNRG),
Infected patients will be invited to participate in this study, written informed consent will be obtained and recruited for a prospective study.
Since the subjects in the study are also children, the consent of a legally authorized representative will be sought.

4.3 Case definition of Chagas' disease:

T. cruzi infection will be diagnosed as follows:

- < 8 months of age: Parasitemia identified by microhematocrit (MH) procedure
- 8 months of age or older: CS, at least two tests must be reactive and PCR positive.

4.4 Studies description

Infected patients attending treatment clinics (see above) following routine clinical, laboratory and cardiological examination, (electrocardiogram, echocardiogram) before receiving treatment will be invited to participate in this study.
If agreed, written informed consent will be obtained. In the case of children the consent of a legally authorized representative will be sought.

From each included patients

- Two milliliters of blood from children and 5 ml of blood from adults will be obtained and identified by date and serial number.
- For the determination of F2/3 and anti-idiotype antibodies serum samples that have been obtained for CS as part of routine diagnosis will be used. F2/3 tests it will be done in the Servicio de Parasitología y Chagas (HNRG).

Subject recruitment will continue until 90 children participants are included (recruitment 12-18 months).

4.5 Follow-up

The patients will be followed up according the routine case management:

- Symptomatology inquiry and physical examination on day 0, and pre defined follow up visits.
- Parasitology evaluation: Parasitemia by MH in less than six months old children at weekly intervals until becoming negative.
- CS after treatment every 3 months during the first year and later, every 6 months.
- Clinical laboratory: hematology, liver and renal function at diagnosis, at 1, and 2 months during treatment.

For the proposed study:

- For those patients that have consented the volume of blood extracted at each visit will be increased (see 4.7 *Blood collection and sampling schedule*) to perform PCR. Consent to determine anti-F2/3 and anti-idiotype antibodies will be determinate in serum samples obtained at time of diagnosis, will be requested. and during routine follow up visits.

(See 4.6 -Timetable for clinical and parasitological)

4.6 Timetable for clinical and parasitological assessment

	Preentry / Entry visit	On-therapy visit	On-therapy visit	End of therapy visit	Post-therapy visit	Post-therapy visit	Post-therapy visit	Post-therapy visit	Post-therapy visit	Post-therapy visit	Post-therapy visit	Post-therapy visit
	Visit 0	Visit 1	Visit 2	Visit 3	Visit 4	Visit 5	Visit 6	Visit 7	Visit 8	Visit 9	Visit 10	Visit 11
Study day	-15d / 0 d	7d	1m	2m	5m	8m	11m	14m	20m	26m	32m	36m
Informed consent	X											
Demographics	X											
Inclusion/exclusion criteria	X											
Physical examination	X	X	X	X	X	X	X	X	X	X	X	X
Blood sampling for												
Parasitemia (MH)*	X	X	X									
CS	X	X	X	X	X	X	X	X	X	X	X	X
PCR	**X**	**X**	**X**	**X**		**X**		**X**		**X**		**X**
Medical history	X											
12-lead ECG	X							X		X		X
Echocardiogram	X							X		X		X
Clinical labs (hematology, liver test)	X	X	X	X								
Adverse event reporting		X	X	X								
Study medication compliance		X	X	X								
Urine pregnancy test #	X											

Activities in "**bold**" indicate activities outside routine patients treatment.
*MH: (Direct parasitological test), blood samples will be drawn in infants younger than 6 months and every 7 days until becoming negative during treatment.
#Urine pregnancy test must be obtained for young females of childbearing potential.

4.7 Blood collection and sampling schedule

Visit	Blood samples for MH[a] ml	Blood samples For Serology [b] ml	Blood samples For PCR [c] ml	Clinical labs (hematology, liver test) ml
0	0.3	1.5	2 i<br>5 ad	1.5
1	0.3	1.5	2 i<br>5 ad	1.5
2	0.3	1.5	2 i<br>5 ad	1.5
3		1.5	2 i<br>5 ad	1.5
4		1.5	2 i<br>5 ad	
5		1.5	2 i<br>5 ad	
6		1.5		
7		1.5	2 i<br>5 ad	
8		1.5		
9		1.5	2 i<br>5 ad	
10		1.5		
11		1.5	2 i<br>5 ad	

a: MH: Direct parsitological test.
b: blood samples will be drawn until becoming negative in two successive visits.
c: i = infants; ad = adults

4.8 Case Report Form (CRF)

All clinical data will be entered into Patient Record Form (PRF), from where transcription will be made into the CRF. Original Laboratory Data will be attached to the PRF and only the results will be transferred into CRF. Only authorized study staff will be allowed to fill in the information in the CRF. The assignment for this activity is described in the *Authorized Signatory Form*, completed and signed by Principal Investigator and the study staff.

5 SELECTION AND WITHDRAWAL OF SUBJECTS/PATIENTS

5.1 Inclusion Criteria:

Subjects meeting all of the following criteria will be considered for inclusion in the study

- Children and adults of up to 22 years of age both sex with *T. cruzi* infection
- Patients that have not received parasiticidal treatment for Chagas´ disease.
- Detectable parasitemia by MH in younger than 8 months
- Reactive conventional serology to *T.cruzi* or *T.cruzi* PCR in older than 8 months.
- Residing within influence area of the clinical centers.
- Written informed consent accepted.

5.2 Exclusion criteria

Subjects presenting with any of the following will not be included in the study:

- Pre-term infants (<36 weeks gestational age).
- Weight < 2500 g
- Older than 22 years
- Pregnancy, as determined by a pregnancy test.
- Women breast-feeding .
- Treatment with any investigation drug in the last month before study entry.
- Cardiovascular, hepatic, neurologic, endocrine, or other major systemic diseases that would make follow up uncertain .
- Immunocompromised subjects.
- History of drug or alcohol abuse.
- Mental condition rendering the subjects, or the pattern/legally authorized representative unable to understand the nature, scope, and possible consequences of the study, or unlikely to comply with protocol, e.g., uncooperative attitude, inability to return for follow-up visits, or unlikely to complete the study.

5.3 Withdrawal criteria

Subjects may be withdrawn from study for the following reasons.

- At their own request or at the request of their legally authorized representative.
- Non adherence to protocol.
- Pregnancy or serious disease during follow up period.
- The occurrence of serious adverse events that may be related to treatment .

In all cases, the reason for withdrawal or discontinuation from the study must be recorded in the CRF and in the PRF.

6 STUDY PROCEDURE

6.1 Patients Treatment (within normal patient management)

Treatment will follow scheme establish by the clinical centers, i.e.: Benznidazole (RADANIL®, Roche) 100 mg / tablets. Dosage: 5-8 mg/kg/day, 2-3 daily doses for 60 days. The drug will be provided by the National Ministry of Health, Argentina. Medication will be provided by monthly batches. Compliance will be monitored at each visit (See below).

.

6.2 Pre treatment

All infected *T. cruzi* individuals will be informed on the details and intentions of the study. Furthermore the details will also include the significance of their participation to the end of the study. After having cleared all doubts a written informed consent will be obtained.

As part of the normal patient management, a detailed clinical history will be taken followed by general physical examination, evaluation of all systems and laboratory routine.

Specific inquiry will be made and examination done to verify inclusion and exclusion criteria.

- F2/3 and PCR will be performed in addition to general laboratory test (hemograma, liver and renal function test), pregnancy test (HCG test in urine) all women >13 years, MH in infants younger than 6 months and CS.

6.3 Methods

Parasitemia by MH

In infants younger than 6 months 0.3 ml of heparinized blood will be taken for direct parasitemia by MH. The parasitemia test involves the microscopic visualization of parasites at a magnification of X 400 in a sample obtained from the buffy coat of heparinized blood in microhematocrit tubes.

Parasitemia by PCR assay

DNA Preparation: in order to disrupt minicircle chains, blood stabilized in equal volume of 6M HCl-guanidine/0.2M EDTA solution, in a boiling water bath during 15 min.

DNA extraction will be performed using a (1:1) phenol :chloroform mixture.

After centrifugation, the pellet will be rinsed with 500 μl of cold 70% ethanol, centrifuged for 15 min and supernatant will be discarded.

The resulting pellet will be dried for 10 min. at 37ºC and then, re-suspended in 50 μl sterile water for 10 min at 37ºC. Ten μl of the extracted DNA will be used for PCR.

T. cruzi DNA amplification by hot-start PCR (15, 16, 17): minicircle DNA (k-DNA) purified from *T. cruzi* will be amplified using the following primers:

121 (5' AAATAATGTACGGG(T/G)GAGATGCATGA 3')

122 (5'GGTTCGATTGGGGTTGGTGTAATATA 3') generating an amplicon of 330 pdb.

Parasite satellite DNA will also be amplified using the following primers:

Tcz1 (5'CGAGCTCTTGCCCACACGGGTGCT 3')

Tcz2 (5'CCTCCAAGCAAGCAGCGGATAGTTCAGG 3') generating an amplicon of 188 pdb.

Reaction mixture: Taq buffer, 25 mM MgCl2, 2,5 mM DNTPs, Tcz1 o 121 Primer (50 μM), Tcz2 o 122 Primer (50 μM), 10 mg/ml BSA, sterile H_20, 5 U/μl Taq DNA polymerase, 10 μl DNA.

Cycles: two 3-min cycles at 94ºC and 68ºC, respectively; 35 cycles at 72ºC, 94ºC y 68ºC, 1 min each; and a single cycle at 72ºC for 15 min.

Detection of amplified products: each amplified sample (15 μl) will be analysed in a 2% agarose gel in TAE 1 X buffer, containing ethidium bromide at 5 μl per 100 ml agarose. PhiX 174/Hae III or 100 bp ladder will also be included as molecular weight markers.

Conventional Serology (CS)

Patients with 2 reactive serological test will be considered infected. These studies will be carried in Servicio de Parasitología y Chagas (HNRG) . Blood samples for serology will be collected into appropriate tubes and will be centrifuged (within 1 hour) at 1500g for 10 min. Serum will be transferred into one polypropylene labeled tube. The tubes will be immediately put in the freezer at –20ºC pending analysis. All serum samples will be stored in Servicio de Parasitología y Chagas (HNRG). Serum samples will be identified by serial number until used. The date of blood samples must be documented in the case report form.

We will employ:

- Enzyme-linked immunosorbent assay (CHAGAS ELISA®, Wiener Lab). Results will be expressed as the ratio (R) between the optical density value recorded for each serum sample and the cut-off value of the assay. Variations in serological values along the follow-up of patients will be measured in order to verify antibodies levels reduction as consequence of effective chemotherapy.
- Indirect hemagglutination assays (HAI Chagas®, POLYCHACO) and/or particle agglutination (Serodia®, Bayer). Results will be estimated as antilogarithms of the log-transformed mean dilutions. Variations in serological values along the follow-up of patients will be measured in order to verify titers reduction as consequence of effective chemotherapy.

Determination of anti-F2/3 antibodies

Parasites: *T. cruzi* bloodstream trypomastigotes of RA strain will be obtained from infected Rockland mice at the peak of parasitemia. Parasites will be used for infection of VERO cell cultures in Dulbecco´s modified Eagle medium supplemented with 10% fetal calf serum. After 6 days of infection, trypomastigotes will be harvested, extensively washed, and chromatographically purified through a DEAE-Sepharose matrix. This process will be repeated upon obtaining of a parasite density high enough to enable antigen purification.

Purification of F2/3 antigen: the procedure described by Almeida et al. (12) will be used. Purified trypomastigotes will be extracted 3 times with chloroform/methanol/water (5:10:4), centrifuged at 12,000xg, and both the extract (a) and pellet (b) will be dried and treated as follows:

(a) A 3-round fractionation using a Butanol/water (2:1) partition system. The Butanol-soluble fraction is called F1, and the water-soluble fraction, F2 (molecular mass, 74-96 kDa).

(b) A 3-round fractionation using 9% Butanol in water for 4 h at room temperature. The soluble extract corresponds to fraction F3 (molecular mass, 120-200 kDa).

Both F2 and F3 fractions will be combined (F2/3) and chromatographed on an octyl-Sepharose column pre-equilibrated with 5% propan-1-ol in 0.1M ammonium acetate buffer, pH 7.2 (buffer A). The F2/3 glycoconjugates will be eluted with a propan-1-ol gradient (5-70%). Eluted fractions will be used for coating of microELISA plates.

Chemiluminiscent ELISA (CL-ELISA): for detection of serum antibodies directed to α-galactosyl-containing epitopes from *T. cruzi* glycoconjugates, which are specifically recognized by trypanolytic antibodies present in sera from Chagas patients with active infection. The F2/3 antigen diluted in 50 mM, pH 9.6 carbonate-bicarbonate buffer will be immobilized on white, opaque 96-well ELISA microplates, overnight at 4°C. After the free binding sites are blocked with 0.1% bovine seroalbumin, plates will be incubated for 30 minutes at room temperature with 50 μl of patients sera diluted 1:2000 in PBS-0.05% Tween 20. The plates will be washed and incubated with 1:2000 dilutions of biotinylated anti-human IgG and then with a streptavidin-horseradish peroxidase conjugate, for 30 min each at room temperature. Plates will be washed 4 times after each incubation period. The reaction will be revealed after a 5-min incubation in Lumiphos-530 reagent (1:10 in coating buffer). The wells will be exposed to Kodak T-MAT S film placed at the bottom of each plate and, after development, films will be attached to the top of unused plates and introduced in an ELISA microtiter reader for densitometric reading at 492 nm. Results will be expressed as the ratio (R) between the optical density value recorded for each serum sample and the cut-off value (mean of negative controls + 3 SD) of the assay.

Variations in R values along the follow-up of patients will be measured in order to verify reduction in F2/3 antibodies levels as consequence of effective chemotherapy. At different time-points in the follow-

up, the kinetics of reduction and/or disappearance for anti-F2/3 antibodies will be compared with that determined for CS in order to establish differences, in the time, between both markers.

6.4 Study follow-up:

Patients will be followed-up during 3 years.

- Within the patients follow up in addition to the routine clinical and laboratory assessments

Anti-F2/3, and PCR it will be done during and after treatment
(*see 4.6-Timetable for clinical and parasitological assessment and 4.7- Blood collection and sampling schedule*)

6.5 Medication(s)/treatment(s) permitted

Concomitant therapy: No parasiticidal drugs will be allowed while receiving benznidazole.

6.6 Procedures for monitoring patient compliance:

The medical staff will administer the drugs and will emphasize the importance of compliance with dosing schedule.
Compliance will be assessed by counting the number of tablets left at each visits .
The use of tablets will be recorded in the CRF.

7 EFFICACY ASSESSMENT

7.1 Specification of efficacy variable and parameters

Primary parameter

Reduction of anti-F2/3 antibodies titer after treatment related to age of patients.
Reduction of CS antibodies titer after treatment

Secondary parameters

Seronegativization of anti-F2/3 after treatment related to age of patients.
Negativization of PCR or MH after treatment.

8 STATISTICS

8.1 Sample size calculation

Along a period of 3 years, we expect a negativization for anti F2/3 antibodies in 80% of subjects and we expect a negativization for CS antibodies in 50 % of subjects and a drop out rate of 20%, then we will need a sample of 90 infants with a 95% of confidence level and a power of 80%.

8.2 Description of statistical method

Geometric mean titers (and 95% CI) will be estimated for serological values.
Variations in R values along the follow-up of patients will be measured in order to verify reduction and/or disappearance in CS and F2/3 antibodies levels as consequence of effective chemotherapy. At different time-points in the follow-up, the kinetics of reduction and/or disappearance for anti-F2/3 antibodies will be compared with that determined for CS, in order to establish differences between both markers.χ^2 with Fisher`s exact test correction will be used to compare % of reduction or negativization between anti-F2/3 and CS. *t test* will be used to compare the time needed for significative reduction or negativization between anti-F2/3 antibodies vs CS.

Statistical data analysis will be performed using:

- GraphPad Prism version 3.00 for Windows, GraphPad Software, San Diego California USA, www.graphpad.com. Copyright (c) 1994-1999.
- EPI-info 6.04: Spanish version. Centers for Disease Control and Prevention, Atlanta, USA, 1994.

8.3 Significance level:

The significance level of 0.05 is used in this study for sample size and for statistical data analysis.
Any deviation from statistical plan will be described in the final report.

9 DATA HANDLING AND MANAGEMENT

All trial data will be recorded on the CRFs. Only the investigators and authorized co-workers, according to the list of Authorized Signatory Form (ASF), are authorized to make entries on the CRFs. CRFs should be completed in Spanish.

Entries into the CRFs should be made with a black or blue ink ballpoint pen to ensure legibility. Corrections should be made by drawing a single line through the original entry, entering the new value and placing initials and date next to the new entry (follow the procedure in SOP CT 05). Completed CRFs will be dated and signed by the investigator or authorized study personnel.

All clinical data will be entered into PRF (source data) from where transcription will be made into the CRFs. Original lab data will be attached in PRF and only the results will be transferred into CRFs. CRFs will be kept in locker assigned for this study.

After completion of the CRFs by the investigator, The data will be entered into a database where computer checks are used to identify selected protocol violations and data errors. Alternately, this can be done manually. Requests for clarifications or correction will be sent to the investigator, if necessary. Statistical analysis will be carried out after all enquiry have been done and data has been locked.

10 ETHICAL ASPECTS

10.1 Approval by the Ethical Committee

The protocol of this study will need approval from the local Ethical Committee it is initiated. The Principal Investigator will submit the study protocol with all-necessary attachments and documentation to these authorities. A copy of the written approval from local ethics committee will be sent to PRD/TDR, the product manager will then submit the proposal to SCRIHS for final approval.

10.2 Subject Information and Informed Consent

(See 17.1, 17.2)

a. Each subject will receive information form and will be explained in presence of witness if required. Sufficient time will be given to the subject to decide whether or not to participate in the study.
b. Participants will be given the opportunity to inquire about details of the study and any questions regarding the study will be answered.
c. Principal Investigator will ensure that the consent form has been signed and dated by the subject, physician and witness.
d. If required, an independent witness will attend the information session and sign and date the consent form prior to the study entry of the subject.
e. Investigator will ensure that the written information and the consent form are revised when required by an amendment to the clinical trial protocol.

Before entering the study, each subject will give a written informed consent. Consent will be verbal for the patients who are illiterate, in this case witness will be required, and the witness will then sign the informed consent form. By signing this protocol, the investigator assures PRD/TDR that informed consent will be obtained and that the consent form and consent information to be used, will be submitted to the Ethical Committee charged of approving the conduct of the study.

10.3 Confidentiality

Subjects' names will not be supplied to the sponsor. Only the subject number and subject initials will be recorded in the case report, and if the subject name appears on any other document it must be obliterated before a copy of the document is supplied to the sponsor. The samples will be coded by the

first letter of the name and the first letter of surname plus day, month and year of birth (dd/mm/yy). Study findings stored on a computer will be stored in accordance with local data protection laws. The subjects will be told that representative of the sponsor local monitor and regulatory authorities may inspect their medical record to verify the information collected and that all personal information made available for inspection will be handled in strictest confidence.

The investigator will maintain a personal subject identification list to enable records to be identified.

11 REFERENCES

1. Tratamiento etiológico de la enfermedad de Chagas. Document OPS/HCP/HCT/140/99.
2. Moya P, Paolaso R, Blanco S *et al*, Tratamiento de la enfermedad de Chagas con Nifurtimox durante los primeros meses de vida. Medicina (Bs As), 45: 553-558, 1985.
3. Freilij H , Altcheh J. Congenital Chagas' disease: Diagnostic and clinical aspects. Clin Infect Dis, 21: 551-5, 1995.
4. Freilij H., Altcheh J., Respuesta terapéutica al nifurtimox en pacientes de edad pediátrica con enfermedad de Chagas crónica. Rev Pat Trop (Brazil), 27: 17-19, 1998.
5. Galvâo L, Nunes R, Cançado J, Brener Z, Krettli A. Lytic antibody titre as a means of assessing cure after treatment of Chagas disease: a 10 years follow-up study. Trans Roy Soc Trop Med Hyg, 87: 220-223, 1993.
6. Krettli AU, Cançado JR, Brener Z. Effect of specific chemotherapy on the levels of lytic antibodies in Chagas disease. Trans R Soc Trop Med Hyg, 76 (3): 334-340, 1982.
7. Krettli, AU. Diagnosis of *Trypanosoma cruzi* chronic infections in humans: Usefulness of the complement regulatory protein antigens and lytic antibodies in the control of cure. Mem Inst Oswaldo Cruz, 94:301-304, 1999.
8. Gazzinelli R, Galvao L, Krautz G y col. Use of *Trypanosoma cruzi* purified glycoprotein (GP57/51) or trypomastigote shed antigens to assess cure for human Chagas disease. Am J Trop Med Hyg, 49: 625-635, 1993.
9. Almeida, IC, Covas, DT, Soussumi, LMT, Travassos, LR. A highly sensitive and specific chemiluminescent enzyme-linked immunosorbent assay for diagnosis of active *Trypanosoma cruzi* infection. Transfusion 37:850-7, 1997..
10. Krautz, GM, Kissinger, JC, Krettli, AU. The targets of the lytic antibody response against *Trypanosoma cruzi*. Parasitol. Today 16: 31-34, 2000.
11. Almeida, IC, Krautz, GM, Krettli, AU, Travassos, LR. Glycoconjugates of *Trypanosoma cruzi*: A 74kD antigen of trypomastigotes specifically reacts with lytic anti-γ-galactosyl antibodies from patients with chronic Chagas disease. J Clin Lab Anal 7:307-16, 1993.
12. Altcheh J, Corral R, Biancardi M, Freilij H. Anticuerpos anti F2/3 como marcador de curación en niños con infección congénita por *Trypanosoma cruzi*. Medicina (Buenos Aires). 2003. In press.
13. Altcheh J, Burgos J, Minervini V, Freilij H, Levin M, Schijman A. Evaluation of polymerase chain reaction of *T .cruzi* DNA as an early marker of cure in pediatric Chagas disease patients under tripanocidal therapy. 44 th Meeting of the American Society of Tropical Medicine and Hygiene, Washington, USA, 1999.
14. Sadigursky M, von Kreuter BF, Ling P, Santos-Buch Ch. Associated of elevated anti-sarcolemma, anti-idiotype Antibody expression of Chronic Chagas Myocarditis. Circulation 80:1269-1276, 1989
15. Sturm N, Degrave W, Morel C et al. Sensitive detection and schizodeme classification of *T. cruzi* cells by amplificaction of kinetoplastid minicircle DNA sequence: use in diagnosis of Chagas disease. Mol. Biochem. Parasitol 1989; 33: 205-14.
16. Avila HA, Sigman DS, Cohen LM, et al. Polymerase chain reaction amplification of *Trypanosoma cruzi* kinetoplast minicircle DNA isolated from whole blood lysates: diagnosis of chronic Chagas' disease. Mol Biochem Parasitol 1991; 48(2):211-21.
17. Schijman AG, Vigliano C, Burgos JM, et al. Early diagnosis of recurrence of *Trypanosoma cruzi* infection after heart transplantation of a chronic Chagas´ disease patient. J H L Transp. 2000; 19:1114-7

12 ABBREVIATIONS USED

CS	Conventional serology
MH	Microhematocrit test
CoML	Complement mediated lysis
PCR	Polymerase Chain Reaction
HAI	Indirect Hemagglutination
ELISA	Enzyme-linked-immuno-assay

13 TENTATIVE PLAN OF ACTION

13.1 Time Schedule in Months

Step	0-6	7-12	13-18	19-24	25-30	31-36
Clinical study & Serology	—	—	—	—	—	—
Preparation of F2/3 Antigen		—	—	—		
PCR	—	—	—	—	—	—
ELISA for F2/3 and Anti-idiotype					—	—

14 CONSENT FORM (Local Language)

14.1 Consentimiento de pacientes adultos para participar en proyecto

Servicio Parasitología y Chagas. Hospital de Niños Ricardo Gutiérrez.

Validación de los anticuerpos anti-F2/3 como marcadores de curación en pacientes pediátricos y adultos infectados con *Trypanosoma cruzi* tratados con benznidazol.

Fecha..........................

Investigador principal: Héctor Freilij
Organización: Servicio de Parasitología y Chagas, Hospital de Niños R. Gutiérrez
Sponsor: Organización Mundial de la Salud. Programa Especial para Investigación y Entrenamiento en Enfermedades Tropicales (TDR).

Nosotros somos médicos que atendemos pacientes y trabajamos en el estudio y tratamiento de la enfermedad de Chagas tanto en niños como en adultos.

Objetivo:
Descubrir nuevos métodos para seguir el tratamiento y así saber mejor y más rápido cuando se haya logrado cura.

Procedimientos:
Como usted sabe ha sido diagnosticado que tiene la enfermedad de Chagas la cual ha podido ser adquirida por la picadura de la vinchuca, durante el embarazo o por una transfusión de sangre. Esta infección se produce por un parásito que vive en la sangre llamado *Trypanosoma cruzi*.
Usted está aquí para iniciar el tratamiento habitual con un medicamento llamado benznidazol. Este tratamiento durará 2 meses y casi seguramente se curará. Este medicamento está aprobado por el Ministerio de Salud Pública y se utiliza desde el año 1973. Es importante que usted sepa que este medicamento es bien tolerado a la dosis con la cual lo vamos a tratar. Sin embargo, algunas veces, puede producir náuseas, dolor de cabeza, mareos, cansancio y trastornos digestivos que pueden aparecer al comienzo del tratamiento. En el curso de la primera o segunda semana de tratamiento pueden aparecer reacciones en la piel como pueden ser enrojecimiento, picazón, ronchas. A partir de los 30 días, pueden manifestarse dolores o molestias en brazos y piernas. Si estas molestias fueran intensas y se acompañan de fiebre y manchas, concurra inmediatamente al hospital. El doctor decidirá si puede continuar o suspender el tratamiento.
Se han observado manifestaciones en la sangre con disminución de los glóbulos blancos y plaquetas.
El tratamiento y la medicación será realizada por nosotros en forma gratuita como parte de la atención habitual del paciente con enfermedad de Chagas.
Para saber si está curado, se realizarán controles médicos y exámenes de sangre durante y luego de terminado el tratamiento. Se dice que usted está curado cuando los estudios de sangre (anticuerpos) son negativos; esto suele observarse luego de varios años.

La Organización Mundial de la Salud concidera que este estudio para buscar nuevos anticuerpos que permitan saber más rápidamente si un paciente que recibe tratamiento está curado es sumamente importante.

Por lo tanto, nosotros estamos invitando a todos las personas con la enfermedad de Chagas para que nos permitan usar su sangre en un estudio donde se verifiquen esos nuevos anticuerpos que permitirán mejorar el conocimiento sobre la enfermedad de Chagas y la atención de los niños y adultos con esta enfermedad.
La decisión de participar o no en este estudio de ningún modo afectará la calidad y el cuidado que le daremos durante el tratamiento.
La participación en este estudio, que durará tres años, no agregará visitas, ni extracciones de sangre adicionales a las que se les realiza a cualquier persona a la que se le da el tratamiento para esta enfermedad .
Para su información los controles médicos y los estudios de laboratorio (Extracción de sangre) se realizan habitualmente con todos nuestros pacientes es el siguiente:
a) durante el tratamiento que dura 2 meses: visitas a los 7, a los 30 y a los 60 días.
b) después de finalizado el tratamiento: cada 3 meses el primer año y luego cada 6 meses hasta completar 3 años.
Durante este estudio, se le solicitará que en las extracciones de sangre que normalmente se hacen en cada visita y que son el equivalente de una cucharada de sopa, en este caso aumentaremos la cantidad a 2 cucharadas soperas. Con esta sangre extra nosotros haremos un estudio nuevo llamado PCR (reacción en cadena de polimerasa) que permite detectar pequeños pedazos del *Trypanosoma cruzi* en la sangre y anticuerpos que nosotros hemos descubierto y creemos que pueden indicar si usted está curado o no más rápidamente. Si usted esta de acuerdo su sangre será guardada en una heladera durante 20 años en el laboratorio de Parasitología y Chagas del Hospital de Niños R. Gutiérrez en Buenos Aires y podrá ser utilizada para estudios posteriores relacionados a la enfermedad de Chagas y posteriormente será eliminada por incineración. Por otro lado debe saber que la cantidad de sangre extraída no provoca ningún inconveniente.

Riesgo y molestias
Las extracciones de sangre ocasionalmente pueden producir un hematoma o generar un leve malestar. La cantidad de sangre que se extrae no suele causar anemia ni problemas en las defensas suyas. Queremos contarle que estas molestias no son diferentes por participar en este estudio.

Beneficios:
Usted no obtendrá ningún beneficio extra por la participación en este estudio. Pero ayudará a poder contestar si estos nuevos anticuerpos permitirán saber más rápidamente si esta curado un paciente que recibe tratamiento.
Este trabajo podría no beneficiar a la sociedad inmediatamente, pero posiblemente sí a las generaciones futuras.

Incentivos:
No recibirán pagos por participar en este estudio.
En el caso que sea necesario usted recibirán el reembolso del costo de traslado al hospital para cada visita.

Confidencialidad
Su identidad será reservada como confidencial, como así los resultados de los estudios realizados mediante la identificación por un código de números y letras. Sin embrago estos datos podrán ser revisados y controlados por personal de la OMS para el control de los mismos. Los resultados de este estudio podrán ser publicados en revistas científicas preservando su identidad.

Derecho a no participar o a retirarse:
Su participación en este estudio es voluntaria. Ud. puede decidir no participar o retirarse cuando Ud. lo considere, sin perder la posibilidad de recibir el tratamiento y el seguimiento adecuado para la enfermedad de Chagas. A su vez usted puede ser retirado del estudio por decisión de su doctor.

Personas con las que puede contactarse:
Si Ud. tiene alguna pregunta referente a este estudio puede comunicarse con:

En Buenos Aires:
- Dr. Héctor Freilij o Dr. Jaime Altcheh al Laboratorio de Parasitología del Hospital de Niños Ricardo Gutiérrez de Buenos Aires. TE. 4964 3093.
- Dr. Tomas Orduna al Servicio de Enfermedades Tropicales del Hospital F.J. Muñiz. Tel 4304-2386

En Santiago del Estero:
- Dr. Rafael Manzur al Instituto de Biomedicina. Tel 421-9118

Consentimiento

Nombre y Apellido del paciente..
No de identificación:.................................
Lugar del Estudio:...
Investigador:...

Yo he sido invitado a participar en este estudio, El propósito de este estudio que es la búsqueda de un nuevo anticuerpo que permitirá saber más rápidamente si esta curado un paciente que recibe tratamiento. Los procedimientos no serán diferentes a los que habitualmente recibe una persona con enfermedad de Chagas con la excepción que se extraerá una mayor cantidad de sangre. De por sí el estudio no genera riesgos adicionales al tratamiento y el seguimiento normal. Entiendo que no hay un beneficio directo para mí, sin embargo mí participación podría ayudar a resolver un problema de salud.
No recibiré pagos por participar en este estudio. En el caso que sea necesario se me reembolsará el costo de traslado al hospital para cada visita.

Yo he leído ó se me ha leído toda la información que se menciona anteriormente. Se me ha dado la oportunidad de preguntar y he recibido las respuestas adecuadas. Yo sé que puedo no participar y esto no significará penalidad ni modificación en la atención que recibiré. Si decido participar puedo retirarme cuando lo considere conveniente sin perder la oportunidad de recibir el tratamiento adecuado. Yo libremente acepto participar en este estudio. Luego de firmar recibiré una copia del formulario.

Nombre del paciente ______________________________
Fecha ____/____/____(dd/mm/aa)
Lugar:
Nombre y firma del tutor:
____________________________________ ___________________

Testigo Fecha y Firma:
______________________________ ____/____/____ (dd/mm/aa)

Usted esta de acuerdo con que:
(i) Su sangre será guardada en una heladera en el laboratorio de Parasitología y Chagas del Hospital de Niños R. Gutiérrez en Buenos Aires SI___, NO___.

(ii) durante: 20 años, SI____, NO____; menos de 20 años (indique cuantos) _____ años.

(iii) Podrá ser utilizada para estudios posteriores solo relacionados a la enfermedad de Chagas, SI____, NO____.

(iv) La sangre sera guardada indicando mi nombre:
SI____, NO_____, prefiero que sea guardada con un codigo que proteje mi confidencialidad

14.2 CONSENTIMIENTO DE NIÑOS PARA PARTICIPAR EN PROYECTO

Servicio Parasitología y Chagas. Hospital de Niños Ricardo Gutiérrez.

Servicio de Enfermedades Tropicales. Hospital F. J. Muñiz.

Instituto de Biomedicina.

Validación de los anticuerpos anti-F2/3 como marcadores de curación en pacientes pediátricos y adultos infectados con *Trypanosoma cruzi* tratados con benznidazol.

Fecha...........................

Investigador principal: Héctor Freilij
Organización: Servicio de Parasitología y Chagas, Hospital de Niños R. Gutiérrez
Sponsor: Organización Mundial de la Salud. Programa Especial para Investigación y Entrenamiento en Enfermedades Tropicales (TDR).

Nosotros somos médicos que atendemos pacientes y trabajamos en el estudio y tratamiento de la enfermedad de Chagas tanto en niños como en adultos.

Objetivo:
Descubrir nuevos métodos para seguir el tratamiento de niños con enfermedad de Chagasa y así saber mejor y más rápido cuando estén curados.

Procedimientos:
Como usted sabe su hijo/a ha sido diagnosticado que tiene la enfermedad de Chagas la cual ha podido ser adquirida por la picadura de la vinchuca, durante el embarazo o por una transfusión de sangre. Esta infección se produce por un parásito que vive en la sangre llamado *Trypanosoma cruzi*.
Usted está aquí para iniciar el tratamiento habitual de su niño con un medicamento llamado benznidazol. Este tratamiento durará 2 meses y casi seguramente su niño se curará. Este medicamento está aprobado por el Ministerio de Salud Pública y se utiliza desde el año 1973. Es importante que usted sepa que este medicamento es bien tolerado a la dosis con la cual vamos a tratar a su niño. Sin embargo, algunas veces, puede producir náuseas, dolor de cabeza, mareos, cansancio y trastornos digestivos que pueden aparecer al comienzo del tratamiento. En el curso de la primera o segunda semana de tratamiento pueden aparecer reacciones en la piel como pueden ser enrojecimiento, picazón, ronchas. A partir de los 30 días, pueden manifestarse dolores o molestias en brazos y piernas. Si estas molestias fueran intensas y se acompañan de fiebre y manchas, concurra inmediatamente al hospital. El doctor decidirá si el niño puede continuar o suspender el tratamiento.
Se han observado manifestaciones en la sangre con disminución de los glóbulos blancos y plaquetas.
El tratamiento de su niño y la medicación será realizada por nosotros en forma gratuita como parte de la atención habitual del paciente con enfermedad de Chagas.
Para saber si está curado, se realizarán controles médicos y exámenes de sangre durante y luego de terminado el tratamiento. Se dice que su niño está curado cuando los estudios de sangre (anticuerpos) son negativos; esto suele observarse luego de varios años.
La Organización Mundial de la Salud concidera que este estudio para buscar nuevos anticuerpos que permitan saber más rápidamente si un paciente que recibe tratamiento está curado es sumamente importante.
Por lo tanto, nosotros estamos invitando a todos los niños con la enfermedad de Chagas para que nos permita usar su sangre en un estudio donde se verifiquen esos nuevos anticuerpos que permitirán mejorar el conocimiento sobre la enfermedad de Chagas y la atención de los niños y adultos con esta enfermedad..

La decisión de que su niño participe o no en este estudio de ningún modo afectará la calidad y el cuidado que le daremos a su niño durante el tratamiento.
La participación en este estudio, que durará tres años, no agregará visitas, ni extracciones de sangre adicionales a la que recibe cualquier niño que esté en tratamiento para esta enfermedad.
Para su información los controles médicos y los estudios de laboratorio (Extracción de sangre) se realizan habitualmente con todos nuestros pacientes es el siguiente:
a) durante el tratamiento que dura 2 meses: visitas a los 7, a los 30 y a los 60 días.
b) después de finalizado el tratamiento: cada 3 meses el primer año y luego cada 6 meses hasta completar 3 años.
Durante este estudio, se le solicitará que en las extracciones de sangre que normalmente se hacen en cada visita y que son el equivalente de una cucharada de postre, en este caso aumentaremos la cantidad a una cucharada sopera. Con esta sangre extra nosotros haremos un estudio nuevo llamado PCR (reacción en cadena de polimerasa) que permite detectar pequeños pedazos del *Trypanosoma cruzi* en la sangre y anticuerpos que nosotros hemos descubierto y creemos que pueden indicar si su niño está curado o no más rápidamente. La sangre será guardada en una heladera durante 20 años en el laboratorio de Parasitología y Chagas del Hospital de Niños R. Gutiérrez en Buenos Aires y podrá ser utilizada para estudios posteriores relacionados a la enfermedad de Chagas y posteriormente será eliminada por incineración.
Por otro lado debe saber que la cantidad de sangre extraída no provoca ningún inconveniente.

Riesgo y molestias
Las extracciones de sangre ocasionalmente pueden producir un hematoma o generar un leve malestar. La cantidad de sangre que se extrae no suele causar anemia ni problemas en las defensas de su hijo/a. Queremos contarle que estas molestias no son diferentes por participar en este estudio.

Beneficios:
Su hijo no obtendrá ningún beneficio extra por la participación en este estudio. Pero ayudará a poder contestar si estos nuevos anticuerpos permitirán saber más rápidamente si esta curado un paciente que recibe tratamiento.
Este trabajo podría no beneficiar a la sociedad inmediatamente, pero las generaciones futuras posiblemente se beneficiarán.

Incentivos:
No recibirán pagos por participar en este estudio.
En el caso que sea necesario ustedes recibirán el reembolso del costo de traslado al hospital para cada visita.

Confidencialidad
La identidad de su hijo será reservada como confidencial, como así los resultados de los estudios realizados mediante la identificación por un código de números y letras. Sin embrago estos datos podrán ser revisados y controlados por personal de la OMS para el control de los mismos. Los resultados de este estudio podrán ser publicados en revistas científicas preservando su identidad.

Derecho a no participar o a retirarse:
Su participación en este estudio es voluntaria. Ud. puede decidir que su hijo no participe o retirarlo cuando Ud. lo considere, sin perder la posibilidad de que el niño reciba el tratamiento y el seguimiento adecuado para la enfermedad de Chagas. A su vez el niño puede ser retirado del estudio por decisión de su doctor.

Personas con las que puede contactarse:
Si Ud. tiene alguna pregunta referente a este estudio puede comunicarse con:

En Buenos Aires:
- Dr. Héctor Freilij o Dr. Jaime Altcheh al Laboratorio de Parasitología del Hospital de Niños Ricardo Gutiérrez de Buenos Aires. TE. 4964 3093.
- Dr. Tomas Orduna al Servicio de Enfermedades Tropicales del Hospital F.J. Muñiz. Tel 4304-2386

En Santiago del Estero:
- Dr Rafael Manzur al Instituto de Biomedicina. Tel 421-9118

Consentimiento

Nombre y Apellido del paciente..
No de identificación:.................................
Lugar del Estudio:...
Investigador:...

Mi niño ha sido invitado a participar en este estudio, El propósito de este estudio que es la búsqueda de un nuevo anticuerpo que permitirá saber más rápidamente si esta curado un paciente que recibe tratamiento. Los procedimientos no serán diferentes a los que habitualmente recibe un niño con enfermedad de Chagas con la excepción que se extraerá una mayor cantidad de sangre. De por sí el estudio no genera riesgos adicionales al tratamiento y el seguimiento normal. Entiendo que no hay un beneficio directo para mi niño, sin embargo su participación podría ayudar a resolver un problema de salud.
No recibiré pagos por participar en este estudio. En el caso que sea necesario se me reembolsará el costo de traslado al hospital para cada visita.

Yo he leído ó se me ha leído toda la informa que se menciona anteriormente. Se me ha dado la oportunidad de preguntar y he recibido las respuestas adecuadas. Yo sé que puedo no participar y esto no significará penalidad ni modificación en la atención que recibirá el niño. Si decido que el niño participe puedo retirarlo cuando lo considere conveniente sin perder la oportunidad de que reciba tratamiento adecuado. Yo libremente acepto que el niño participe en este estudio. Lugo de firmar recibiré una copia del formulario.

Nombre del paciente ______________________________
Fecha ____/____/____(dd/mm/aa)
Lugar:
Nombre y firma del tutor:
_____________________________________ ___________________

Testigo Fecha y Firma:
______________________________ ____/____/____(dd/mm/aa)

Usted esta de acuerdo con que:
(i) La sangre de su niño será guardada en una heladera en el laboratorio de Parasitología y Chagas del Hospital de Niños R. Gutiérrez en Buenos Aires SI___, NO___.
(ii) durante: 20 años, SI____, NO____; menos de 20 años (indique cuantos) _____ años.
(iii) Podrá ser utilizada para estudios posteriores solo relacionados a la enfermedad de Chagas, SI____, NO____.
(iv) La sangre sera guardada indicando su nombre:
SI____, NO_____, prefiero que sea guardada con un codigo que proteje su confidencialidad
