# Supplementary material for: Longitudinal follow up of serological response in children treated for Chagas disease
Source: PLoS Negl Trop Dis. 2019 Aug 29;13(8):e0007668. doi: 10.1371/journal.pntd.0007668 (PMC6715178; doi:10.1371/journal.pntd.0007668)
Supplement: S3 File — (DOC) [file pntd.0007668.s003.doc]

**Not included n: 20**

Not consent n: 1

Did not understand study n: 5

Living outside Buenos Aires n: 14

**Elegible patients**

**n: 127**

**Included patients**

**n: 107**

Start treatment

**Completed treatment n: 91**

## Desertion n: 16

Lost during monitoring n: 8

Side effects n: 7

Patient’s decision n: 1

## 3 years of follow up

## n: 76

Start treatment

## Lost during follow-up n: 17

Lost n: 13

Patient decision n: 2
